# Reimagining the joint task force core competency framework for rural and frontier clinical research professionals conducting hybrid and decentralized trials

**DOI:** 10.3389/fphar.2023.1309073

**Published:** 2023-12-21

**Authors:** Jamie M. Besel, Elizabeth A. Johnson, Jiahui Ma, Becky Kiesow

**Affiliations:** ^1^ Billings Clinic, Collaborative Science and Innovation, Billings, MT, United States; ^2^ Mark and Robyn Jones College of Nursing, Montana State University, Bozeman, MT, United States; ^3^ Biomedical Innovation for Research and Development Hub, Montana State University, Bozeman, MT, United States; ^4^ Norm Asbjornson College of Engineering, Montana State University, Bozeman, MT, United States; ^5^ Billings Clinic, Diabetes Research, Billings, MT, United States

**Keywords:** clinical trial enrollment, rural, frontier, decentralized trials, clinical research professional, joint task force competency framework, clinical research workforce

## Abstract

**Introduction:** Clinical research professionals (i.e., clinical research assistants, clinical research nurses, clinical research coordinators, etc.), as outlined by the Joint Task Force (JTF) Core Competency Framework, are highly trained to support the breadth of clinical trial operations and manage participant care. Clinical research professionals are uniquely equipped with a scope of practice that permits product administration, participant assessments, and data management. As clinical trials grow in complexity and their management expands beyond traditional, site-based operations models to decentralized and/or hybrid models, the need becomes great to ensure adequate staffing. However, rural hospitals frequently lack the research staff or patient recruiters that would allow them to support decentralized clinical trials across a sizeable rural geographic demographic.

**Methods:** This paper examines the contributory factors of the clinical research professional workforce contraction and response efforts at professional and organizational levels within a large, Magnet-designated healthcare system in the rural northwestern United States. Perspectives are shared on adapting the Core Competency Framework to reflect the unique strengths and opportunities towards decentralized trials in rural regions of the United States and areas of priority for workforce cultivation and retention. A descriptive survey was used to gather initial data identifying the current research perspectives of healthcare workers working across a rural community. Participants were asked to complete questions about the JTF Competency domains and behavior-based questions.

**Analysis:** Both competency and behavior-based questions were asked and related to roles. These were then cross-referenced using a Rasmussen Ladder system. Descriptive statistics were conducted for sample characteristics, self-reported competency domain questions, and behavior questions.

**Results and discussion:** Survey findings suggest that although healthcare workers and clinical research teams interact, they are unlikely to ask their patients to participate in research. Based on the limited response rate, results suggest that better education throughout the rural community could benefit from decentralized research efforts. Increased use of technology was also highlighted as an area of interest.

## Introduction

The complexity and number of clinical trials have increased significantly over the past several years. Between 2010 and 2020, there was a 300% increase in the number of clinical trials registered on ClinicalTrials.gov (U. S National Library of Medicine, 2023). Clinical research professionals (CRPs) are healthcare professionals highly trained to support most of the day-to-day clinical trial activities, as outlined by the Joint Task Force (JTF) Core Competency Framework (Sonstein and Jones, 2018; Sonstein et al., 2022). They are the “boots on the ground” clinical trial workforce critical to successful clinical trial procedures in real-life situations (Ibrahim et al., 2022).

As the number and complexity of clinical trials have increased over time, so have the responsibilities of CRPs. Clinical research professionals require foundational knowledge and technical expertise in scientific communications and data management; however, they also need other strengths, such as problem-solving and critical thinking (Baer et al., 2011; Chacon and Janssen, 2021). As their management expands beyond traditional, site-based operations models to decentralized and hybrid models, the need to recruit and retain experienced CRPs is even greater (Ibrahim et al., 2022; Freel et al., 2023). However, the CRP workforce continues to decline rapidly, a problem only compounded in rural and frontier areas of the United States. Rural hospitals frequently lack research staff or patient recruiters that would allow them to support decentralized clinical trials across a sizeable rural geographic demographic (Baquet et al., 2006; Seidler et al., 2014; Iglehart, 2018; Winter et al., 2018; Schmidt et al., 2020; Bharucha et al., 2021).

Decentralized clinical trial (DCT) activities occur at locations other than traditional trial sites; these activities may occur at trial participants’ homes or in local healthcare facilities convenient for trial participants (LaHucik, 2021; DiMasi et al., 2023). In hybrid DCTs, some trial activities involve in-person visits by trial participants to other non-traditional clinical trial sites, such as participants’ homes or virtual meetings (U.S. Food and Drug Administration, 2023). Although general competencies for CRPs have been described in the literature, the competencies unique to decentralized trials in rural areas have not been detailed in the literature nor outlined by governing bodies, leaving a significant gap in supporting the development of this essential research workforce. The CRP profession faces a workforce and diversity shortage (Freel et al., 2023). There is a heterogeneity in CRPs with various levels of education that exist. Clinical research professionals are responsible for a wide variety of trials and may not be specific to one indication, which adds to the complexities of the job. While early-phase research is being conducted in rural areas, the most prevalent trial types consist of Observational, Phase 3, Phase 4, and investigator-initiated pilot studies (Goodson et al., 2022).

Nationally, the clinical research workforce has seen workload and study complexity alterations over the past 15 years, corresponding with a 300% increase in registered clinical trials (U. S National Library of Medicine, 2023). Organizations seeking to become more adaptive and innovative often see that culture change is the most challenging part of the transformation process (Walker and Soule, 2017). Rural healthcare and research sites continue to struggle to maintain and retain adequately trained staff with researchers finding less than 12% of US physicians practice in rural areas (MacQueen et al., 2018).

There is a lack of literature surrounding implementing and adapting the JTF Core Competency Framework among United States rural research-driven healthcare systems. However, studies conducted by Schmidt et al. (2020) and Quilliam et al. (2022) have demonstrated a turn in focus toward those centers that have the outreach capabilities to otherwise under-represented populations. Schmidt et al. (2020) conducted qualitative descriptive interviews with 18 rural research professionals with perspectives including low research visibility in clinical care service environments; misconceptions related to lack of research capacity as a rural organization; and overall lack of knowledge and training due to organizational system structures which impede effective change management. Similarly, Quilliam et al. (2022) conducted a qualitative descriptive study, which included 20 participants who echoed the lack of tailoring training to the rural context of conducting clinical research, particularly ensuring that the training is deemed relevant and easily applied into practice.

This paper examines the contributory factors of the CRP workforce reduction and response efforts at professional and organizational levels within a large, Magnet-designated healthcare organization in the rural northwest United States. Perspectives are shared as to adapting the Core Competency Framework to reflect the unique strengths and opportunities towards decentralized trials in rural states and areas of priority for workforce cultivation and retention.

### About the healthcare organization

Geographically located between the great hospital complexes of Minneapolis and Seattle, our large Magnet-designated healthcare organization is uniquely positioned to perform clinical research throughout this rural region. The healthcare system includes three regional branch clinics and 20 Critical Access Hospitals providing healthcare across a sparsely-populated rural and frontier area of over 162,000 square miles—roughly the geographic size of Ohio, Indiana, Illinois, and West Virginia *combined*. Providers, learners, and all staff across the organization have internal access to support for research activities, including clinical and device trials, investigator-initiated translational research, and quality improvement initiatives. The organization is involved in Phase I, II, III, and IV clinical trials and has over 30 years of experience in health system research. Since the research program’s inception in 1988, our staff have worked with over 75 pharmaceutical companies, offering over 250 clinical research studies to patients.

With 15 full-time employees dedicated to research across three key departments driving the clinical research portfolio, challenges exist in ensuring that change management efforts are rolled out seamlessly. Knowing that 50%–70% of change management efforts fail (Mansaray, 2019), we look towards the JTF Core Competency Framework to support research personnel and expansion efforts. Employing a well thought out system, like the JTF Core Competency Framework, should set our organization up for success as we implement new research functionality in our EHR system.

## Guiding frameworks

### Joint task force core competency framework

Competency frameworks are essential to achieving high institution performance (Sonstein and Jones, 2018; Sonstein et al., 2022). Developed in 2014, The Joint Task Force (JTF) Core Competency Framework intends to align clinical researchers worldwide by using a comprehensive set of competencies expected to aid in building a person’s skillset from basic to advanced levels. Using 8 competency domains and 48 specific competency statements, skills are broken down into three levels—fundamental, skilled, and advanced (Sonstein and Jones, 2018; Sonstein et al., 2020). This framework encompasses the knowledge, skills, and attitudes necessary for conducting clinical research within organizations and creates a roadmap to help develop CRPs. Instead of focusing solely on regulatory compliance, the framework identifies professional competency encompassing a clinical trial’s various aspects. Limitations exist using the framework as it does not consider non-interventional, quasi-experimental, mixed methods, and qualitative studies (Ibrahim et al., 2022). Implementation into the rural healthcare setting will require adaptation to represent the complexity of the work environment and different education levels.

### Rasmussen ladder (risk management framework)

**FIGURE 1:**
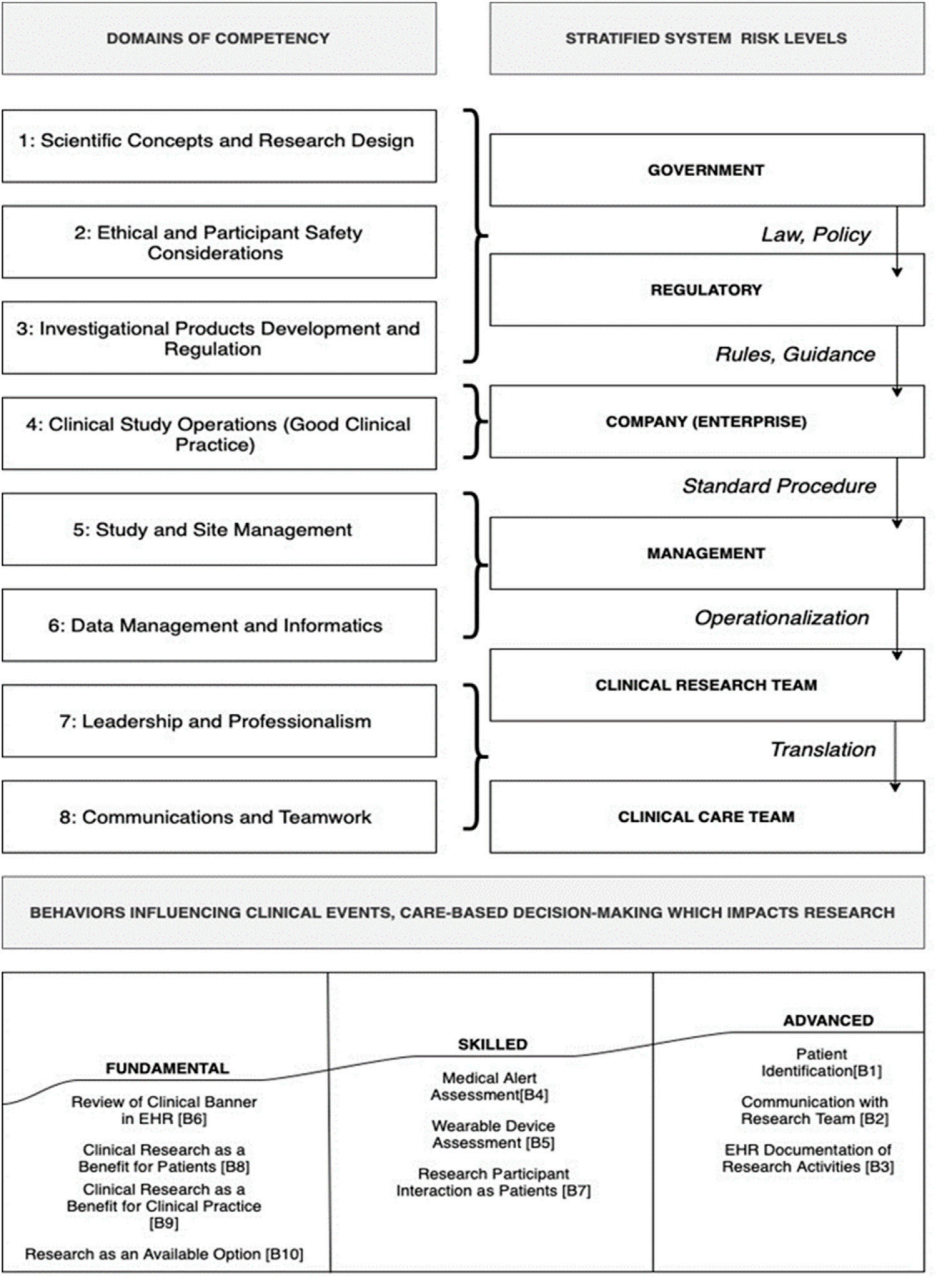
Rassmussen ladder of framework competencies and healthcare system clinical operations.

Given the constant flux in the healthcare industry, there is a continuous appraisal of risk to clinical research operations with the ebb and flow of change at the largest system levels (policy, regulations) and the local systems of research conduct (team collaboration and participant-based encounters). To aid in categorizing and appraising risk across systems levels, organizations employ Rasmussen’s risk management framework (Rasmussen, 1986; Rasmussen, 1997; Brady and Naikar, 2022). The risk management framework (Figure 1) aids in the appraisal of staff behaviors and skill sets as they relate to external factors that may affect the completion of tasks (Rasmussen, 1986; Lintern, 2010). External factors are categorized from mesosystem to microsystem: government (law), regulatory (regulations), company (enterprise), management (plans and policy); and staff (action) (Donovan et al., 2015). These factors as system levels influence one another and thus the ability of an organization and its staff to complete work safely and compliant with oversight entities. As the clinical research industry is highly regulated but varied in its company-specific organization of policies and procedures, the Rasmussen risk management framework was employed in this study to evaluate the organizational enterprise for non-overt influences on quality clinical trial conduct across a wide geographic and cultural range of locations. In conjunction with the JTF Core Competency Framework, the lens of classical risk management modeling gleans insight into how competency level may influence the organizational approach to risk associated with clinical research programs or departments.

### Purpose

The purpose of this study was twofold: 1) explore the self-perceived competence level among the 8 domains of the JTF Core Competency Framework among organizational research and clinical care professionals and 2) examine self-reported frequency in behaviors associated with clinical research risk management. Together, these two aims provide the foundation for understanding contributory factors associated with rural clinical research workforce recruitment and retention. Furthermore, as the rural organization expands the clinical research technological infrastructure across its vast geographical expanse of locations, this study serves as an initial insight into priority areas during the period of change among the risk management attributes. The long-term goal of this research is to iterate an adaptive version of the JTF Core Competency Framework congruent to rural organizations to inform best training and workforce recruitment/retention practices with a focus on decentralized trial conduct.

## Methods

### Design and approach

A descriptive survey design was selected to gather initial, formative data that reflects research and clinical care personnel’s perspectives, behaviors, and beliefs surrounding the core competencies (Kelley et al., 2003). Given the geographical distances between the 23 affiliated facilities, an online survey was selected to promote better reach of the study.

The order of survey questions, significant domains, and the behaviors to align with risk management attributes were selected through listening sessions with key informants from the rural healthcare organization, including a clinical research coordinator and nurse manager. Upon final consensus, the survey was reviewed by the organization’s Privacy and Exemption Committee along with organizational executive leadership for discussion. The risk management attributes were defined and selected based on priority stratifications surrounding the healthcare system: *Government* and *Regulatory* (Macrosystem); *Management* and *Company (Enterprise)* (Mesosystem); *Clinical Care Team- Clinical Events* and *Clinical Research Staff* (Microsystem).

### Sample and recruitment

Upon approval by the Montana State University Institutional Review Board (Protocol #2023-604) and the organization’s Privacy and Exemption Committee, a Qualtrics survey link was internally distributed through the Intranet and via the organization email listserv. Purposeful and snowball sampling was employed, targeting facility locations affiliated with the rural healthcare organization and groups of clinical research professionals, medical leadership, nursing leadership, and administration. Participants were included if 18 years of age or older, proficient in written English, and if they were an employee in either a clinical care or research role with the ability to complete the survey online. The following groups were included in the email recruitment: research personnel, physicians, physician assistants, nurse practitioners, nursing (hospital and clinic), library, laboratory, leadership, pharmacy, and care management.

### Data collection

**FIGURE 2:**
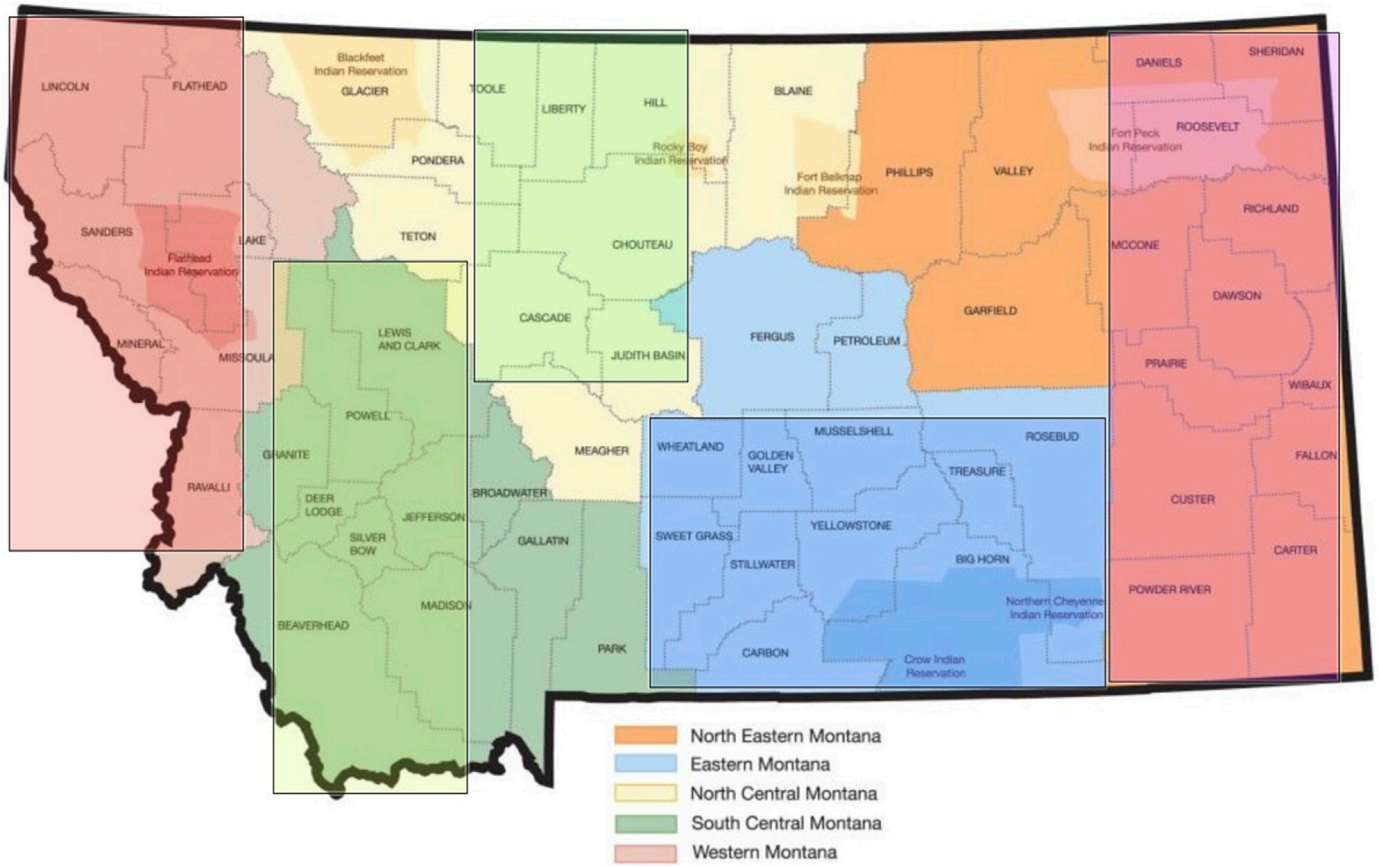
Categorized regional map of Montana for response selection.

Data were collected from August through September 2023 via an anonymized Qualtrics survey link hosted by Montana State University, a research partnering institution for this project. Upon clicking the survey link, a study overview, and a checkbox to indicate consent to proceed to the response fields were provided. Participant demographic and organizational role-based characteristics were first obtained, followed by an interactive regionalized map of the state, which permitted the participant to select the area of state in which their facility was located: Northeastern, Eastern, North Central, South Central, and Western (Figure 2).

An organizational role was requested, which included the categorization of licensed versus non-licensed professionals and those who primarily worked in a research or clinical setting. Participants then proceeded to the JTF Core Competency Framework domain questions, which asked for self-reports of competency level as *fundamental* (can perform the task at an essential level with possible coaching/supervision); *skilled* (can perform the task independently with moderate expertise and high-quality work output); and *advanced* (ability to teach or supervise others with the application of critical thinking and problem-solving). See Table 1 for the listing of domains provided for self-evaluation. Participants used an electronic slider to move their cursor to their self-reported degree of competency with 0 being not competent at all and 100 being fully competent. *Fundamental* skillset was considered 0-50, *skilled* 60-80, and *advanced* 80-100.

**TABLE 1:** JTF competency domains itemized on survey with description.

Domain number and title	Domain description
1: Scientific Concepts and Research Design	Encompasses knowledge of scientific concepts related to the design and analysis of clinical trials
2: Ethical and Participant Safety Considerations	Encompasses care of patients, aspects of human subject protection, and safety in the conduct of a clinical trial
3: Investigational Products Development and Regulation	Encompasses knowledge of how investigational products are developed and regulated
4: Clinical Study Operations (Good Clinical Practice)	Encompasses study management (adverse event identification and reporting, post-market surveillance, and pharmacovigilance), and investigational product handling.
5: Study and Site Management	Encompasses content required at the site level to run a study (financial and personnel aspects). Includes site and study operations (not encompassing regulatory/GCPs)
6: Data Management and Informatics	Encompasses how data are acquired and managed during a clinical trial, including source data, data entry, queries, quality control, and correction and the concept of a locked database
7: Leadership and Professionalism	Encompasses the principles and practice of leadership and professionalism in clinical research
8: Communications and Teamwork	Encompasses all elements of communication within the site and between the site and sponsor, CRO, and regulators. Understanding of teamwork skills necessary for conducting a clinical trial.

After self-evaluation of competency across the eight framework domains, participants then responded to behavior-based questions noted in Table 2 which align with the framework domains and the six attributes of risk management adapted to the context of clinical research, as determined by the organization’s research professionals. Some domains and attributes were measured more than others due to the significance placed by the organization on these elements of research personnel competency, including Domain 8 (*Communications and Teamwork*) and Domain 2 (*Ethical and Participant Safety Considerations*). Doubly measured attributes included: *Clinical Care Team- Clinical Event*, *Management*, and *Company (Enterprise)*. Participants provided their responses in Likert format, which spanned *Never (1), Sometimes (2), Half the Time (3), Most of the Time (4),* and *Always (5).*

**TABLE 2:** Survey questions examining behaviors related to core competency framework and risk management.

Behavior-based question	Alignment with domain (itemized)	Risk management attributes
1: I ask the patient if they are part of research or clinical trial	4: Clinical Study Operations (Good Clinical Practice)	Company (Enterprise)
2: I communicate with research teams to develop or implement the patient’s plan of care	8: Communications and Teamwork	Clinical Research Staff
3: I document in the Electronic Health Record that the patient is part of research or clinical trial	6: Data Management and Informatics	Regulatory
4: I assess for medical alert bracelets on each patient	2: Ethical and Participant Safety Considerations	Clinical Care Team- Clinical Events
5: I assess for wearable devices on each patient	3: Investigational Products Development and Regulation	Clinical Care Team- Clinical Events
6: I review the patient’s banner in the Electronic Health Record for each patient as part of handoff	8: Communications and Teamwork	Clinical Care Team- Clinical Events
7: I interact with clinical trial/research participants as patients	5: Study and Site Management	Management
8: I believe that clinical research benefits my patients	2: Ethical and Participant Safety Considerations	Government
9: I believe that clinical research benefits my clinical or leadership practice	7: Leadership and Professionalism	Management
10. I believe clinical research is important to make available to my patients	1: Scientific Concepts and Research Design	Company (Enterprise)

Upon completion of the behavior questions, the participant had the option to include their e-mail address to be entered into a raffle for one $150 Amazon electronic gift card. An additional opportunity for iterative, focus group feedback and organizational report-out pertaining to these competencies was offered at the end of the survey; participants inputted their e-mail address in the corresponding field if they were interested in continuing to share their perspectives related to clinical research core competencies at a later date.

### Data management and analysis

Data were stored in a secure, encrypted repository hosted by Montana State University. Raw data downloaded from Qualtrics was then placed in restricted-use folders to protect the privacy and confidentiality of participants. Folder access was controlled and limited to only those researchers identified on this project to the Montana State University Institutional Review Board. Data to be used for analysis was kept separately from raw data within the repository.

Survey responses were initially organized using Excel and then coded for analysis using R programming language (Version R-4.30) (R Core Team, 2013). Descriptive statistics were then conducted for sample characteristics (licensure status and role) as well as the self-reported competency domain questions and behavior questions (Table 3). Fisher’s exact tests were conducted given the small, pilot sample size. Odds ratios were then calculated for licensure status which was significantly associated with behavior ratings and competency levels (fundamental, skilled, advanced, Table 4).

**TABLE 3:** Sample characteristics, competency and behavior ratings.

Demographic information										
		Licensed					Non-Licensed			
Licensure status		13 (61.9)					8 (38.10)			
		Clinical					Research			
Role		17 (80.95)					4 (19.05)			
**Competency Rating**										
	Fundamental			Skilled		Advanced			Missing	
Domain 1	5 (23.81)			4 (19.05)		10 (47.62)			2 (9.25)	
Domain 2	2 (9.25)			3 (14.29)		11 (52.38)			5 (23.81)	
Domain 3	6 (28.57)			4 (19.05)		6 (28.57)			5 (23.81)	
Domain 4	3 (14.29)			4 (19.05)		9 (42.86)			5 (23.81)	
Domain 5	6 (28.57)			4 (19.05)		7 (33.33)			4 (19.05)	
Domain 6	3 (14.29)			2 (9.25)		8 (38.10)			8 (38.10)	
Domain 7	3 (14.29)			5 (23.81)		8 (38.10)			5 (23.81)	
Domain 8	3 (14.29)			2 (9.25)		8 (38.10)			8 (38.10)	
**Behavior Question Rating**										
	Never		Sometimes		Half Time	Most Time		Always		Missing
Behavior 1	6 (28.57)		2 (9.25)		-	2 (9.25)		1 (4.76)		10 (47.62)
Behavior 2	5 (23.81)		2 (9.25)		1 (4.76)	1 (4.76)		3 (14.29)		9 (42.86)
Behavior 3	5 (23.81)		3 (14.29)		-	1 (4.76)		3 (14.29)		9 (42.86)
Behavior 4	3 (14.29)		4 (19.05)		-	2 (9.25)		1 (4.76)		11 (52.38)
Behavior 5	2 (9.25)		3 (14.29)		-	4 (19.05)		2 (9.25)		10 (47.62)
Behavior 6	1 (4.76)		2 (9.25)		-	-		7 (33.33)		11 (52.38)
Behavior 7	3 (14.29)		6 (28.57)		-	1 (4.76)		3 (14.29)		8 (38.10)
Behavior 8	-		-		-	5 (23.81)		9 (42.86)		7 (33.33)
Behavior 9	-		-		1 (4.76)	4 (19.05)		9 (42.86)		7 (33.33)
Behavior 10	-		-		1 (4.76)	3 (14.29)		10 (47.62)		7 (33.33)

* The value in each cell: Frequency (Relative Frequency %).

**TABLE 4:** Domains and competency behaviors examined by licensure status and role.

	Licensure status	Role
Domain 1	0.22	0.12
Domain 2	0.10	0.73
Domain 3	0.33	1.00
Domain 4	0.29	0.76
Domain 5	0.35	0.37
Domain 6	1.00	1.00
Domain 7	0.01*	0.33
Domain 8	1.00	1.00
Behavior 1	1.00	0.45
Behavior 2	1.00	0.23
Behavior 3	1.00	0.18
Behavior 4	0.60	0.30
Behavior 5	0. 36	1.00
Behavior 6	0.10	0.30
Behavior 7	0.54	0.15
Behavior 8	1.00	1.00
Behavior 9	1.00	0.23
Behavior 10	1.00	0.63

* Denotes significance *p* < 0.05; Missing values were excluded in the Fisher’s exact test.

### Ethical considerations

This study was deemed minimal risk by the Montana State University Institutional Review Board. To maintain the privacy and confidentiality of those participating, data that may easily identify organization personnel were not collected, such as demographics and facility location of employment. Given the rural and micropolitan settings where these facilities are located, there is a high degree of risk of identifying participants due to the low overall sample population across the organization’s enterprise. As such, professional roles were delineated by the presence or absence of licensure and daily role function as majority research-based or clinical care. Emails provided for the Amazon electronic gift card raffle were destroyed after the raffle was completed to protect further the identity of those who completed the survey.

## Results

A descriptive survey was used to gather initial data identifying the current research perspectives of healthcare workers working across a rural community in the northwest. Participants were asked to complete questions about the JTF Competency domains and behavior-based questions. Of the 21 respondents, more participants were licensed (61.9%) and enrolled in a clinical work setting (80.95%). Although healthcare workers interact with clinical research participants (Behavior 7) and believe clinical research is important to make available to patients (Behavior 10), they are unlikely to ask the patient to participate in a research study and work with the research team (Behaviors 1 and 2). Nearly half (47.62%) of participants identified an advanced competency skill in Domain 1(Scientific Concepts and Research Design) and 52.38% in Domain 2 (Ethical and Participant Safety Considerations). Conversely, participants did not feel as competent with study and site management (Domain 5) and clinical study operations (Domain 3).

**FIGURE 3:**
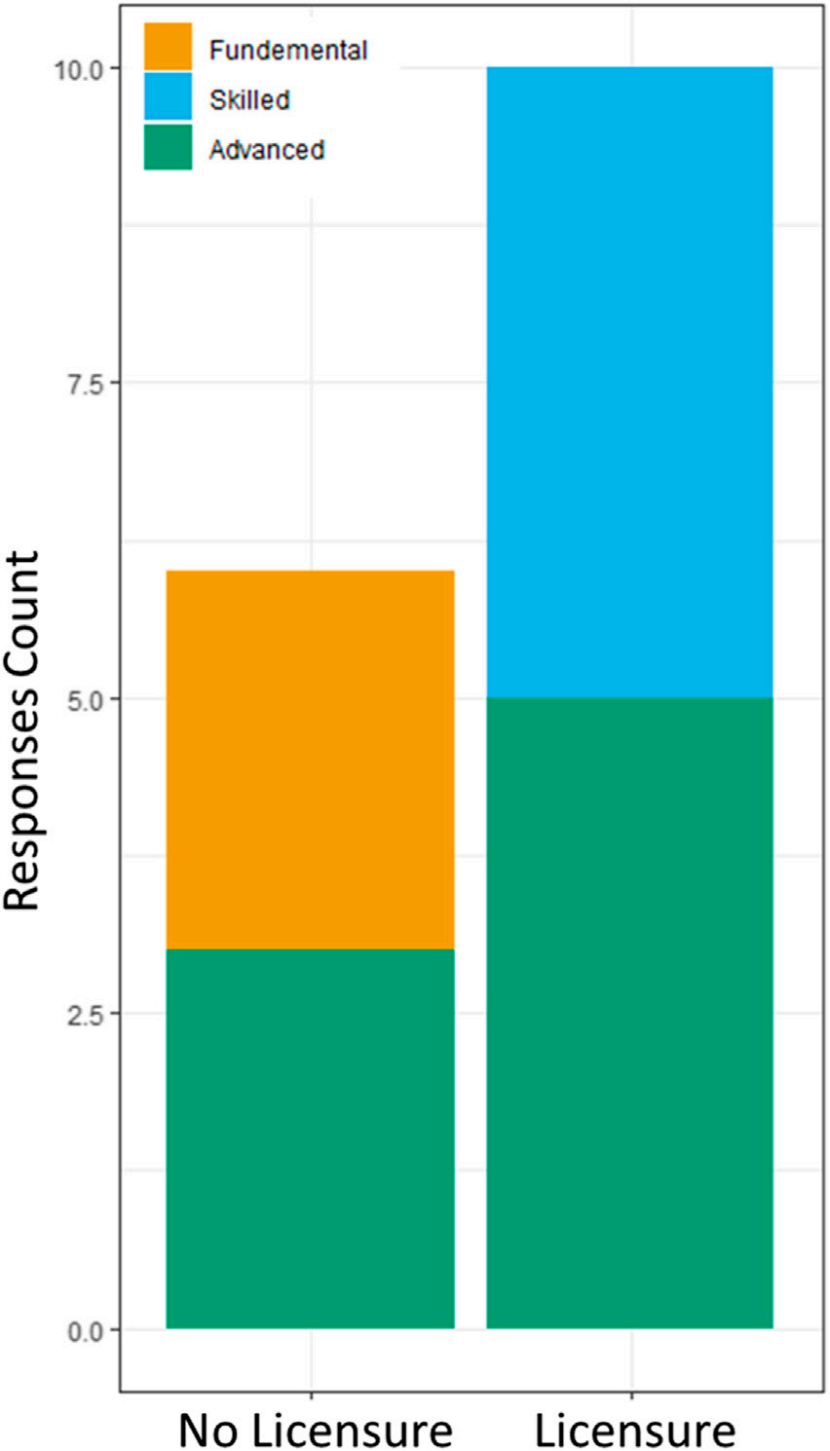
Domain 7 (leadership and professionalism) response count by licensure.

Fisher’s exact test revealed that licensure status was significantly associated withDomain 7 (Leadership and Professionalism) rating (Table 4). Participants with skilled (OR = 9.88e+16) and advanced (OR = 5.24e+08) ratings for Domain 7 had a higher chance to be licensed compared to participants with fundamental leadership and professionalism skills (Figure 3).

## Discussion

Through an iterative refinement process with participants and other key stakeholders, this study explored initial, formative data that reflects the perspectives, behaviors, and beliefs of research and clinical care personnel surrounding the JTF core competencies. This paper examined the contributory factors of the CRP workforce reduction and response efforts across professional and organizational levels at a large healthcare organization in the rural northwest. Using the JTF Framework allows organizations to build necessary research skill sets transferable to frontline healthcare workers in the rural healthcare setting. Employees are encouraged to adapt their behaviors and mindset to ensure patient safety, career growth, and collaboration between those in the field. Similar to the current literature, albeit limited, our survey findings suggest that although healthcare workers interact with clinical research participants, they are unlikely to ask the patient to participate in a research study and work with the research team. As suggested by MacQueen et al., 2018, rural healthcare and research sites continue to struggle to maintain and retain adequately trained staff with researchers. Although only 21 participants completed the survey in our study, this suggests that our research teams could better educate the community about available research opportunities. In a qualitative study by Schmidt et al. (2020), there was an overall lack of knowledge and training due to organizational system structures, further impeding effective change management. Outreach efforts should be investigated further as a solution to building awareness and trust in the rural healthcare setting. The advancement of CRP talent among rural populations would aid in all aspects of decentralized trials and could strengthen the field by ensuring capable research staff are prepared to address the unique complexities inherent in rural healthcare. With the increase of technology in studies, there is an opportunity to engage more with our rural communities, although limitations present themselves due to access issues to modern-day technologies.

Based on the results of this study, the authors recommend further investigation of the competency domains relative to rural decentralized trials and discussions on updating and/or adapting the JTF framework to accommodate rural CRPs who manage decentralized trials. Currently, there is not a risk-based management component that is separated as a priority in the framework as it pertains to decentralized trials in rural areas. As highlighted in the results of this study, providers are not asking patients if they are participating in a clinical trial or evaluating medical devices for their integration in clinical care. This is concerning, especially without the integration of the risk management component. For example, any patient on any given trial could seek care at the organization and be from a different group and/or trial site. Another area to consider is integrative, effective communication via technology. Specifically, how is the organization consistently and accurately messaging the significance of clinical research as a care option or its impact on clinical care delivery? We recommend expanding Domain 8 and potentially creating a separate domain for decentralized/hybrid trials because they necessitate different skillsets and competencies given the variability of resourced environments. Without attention given to this domain, the decentralized model does not work in rural areas. This is because there is a lack of awareness of potential trial participants, no communication, and no adaptation of remote-task skillsets among CRPs. In conjunction with the JTF Core Competency Framework, the lens of classical risk management modeling using Rasmussen’s Framework can glean insight into how competency level may influence the organizational approach to risk associated with clinical research programs or departments. Given the emphasis on clinical trial participant safety in the community setting, adverse event reporting, and shared information exchange of research information pertinent to clinical care, the augmentation of the framework to that of a risk-based organizational model particularly in rural or low-resource settings will aid in the development of responsible trial portfolio expansion and workforce development.

## Limitations

Given the exploratory nature of this initial, formative study, there were noted limitations. While clinical research is an aspect of the organization’s mission, there is no mechanism during the orientation of clinical staff about research opportunities and necessary behaviors that are protective towards research participation in the clinical milieu. The sample size for this study was not statistically powered. However, future research and current activities that amplify awareness of trial opportunities to the enterprise at large will permit statistical analysis generalizable to the region and other rural institutions. Missingness of responses was observed given the voluntariness of each question should the respondent wish not to answer. Given the small number of research-dedicated staff, we could not describe competency by CRP designation (i.e., coordinator, nurse, manager). However, future research will entail focus groups that permit the examination of role-specific competencies and insights. Results from this initial study inform the line of questioning for the focus groups and the educational materials and training necessary for the research portfolio expansion at this healthcare organization.

### Implications for industry sponsors and clinical research workforce

The expanded use of decentralized trial elements and models of research delivery bring a heightened need to evaluate workforce allocation and labor optimization to ensure responsible, compliant conduct outside the traditional research site. In the wake of the COVID-19 pandemic, the site-based clinical research workforce, which includes clinical research nurses (CRNs) and nurse researchers, showed signs of significant contraction (Johnson, 2022). The overall high turnover rate of CRPs, compounded with retirements and increased demand, requires a critical pause and evaluation of how best not only to retain and recruit CRPs but also establish standardization in core competencies, particularly in regions with a reduced pool of candidates (Freel et al., 2023). While sites may conduct a mix of decentralized, hybrid, and traditional research designs, 81.9% of protocols between 2019 and 2020 had at least some decentralized elements about data collection (de Jong et al., 2022). The inclusion of monitoring remote technologies, managing multiple sources of data collection, and mitigating any issues that arise with the technologies requires an expanded CRP skillset. In rural areas where sparse Internet connectivity and potential mistrust of novel technologies may be evident, CRPs must be additionally agile in their appraisal of resources local to the participant to maintain data integrity and device validity.

Recruitment of participants is a costly endeavor that rests on the shoulders of site CRPs. Without a solid organizational structure supporting research activities, recruitment slows and amounts to generalized trial sponsor financial losses of upwards of USD 8 million per day (Thakur and Lahiry, 2021). Financial evaluation of decentralized and traditional trial design has demonstrated that the core factor to cost reduction and participant recruitment success has been the efficiency gained over time from experienced CRPs with the organizational structure in place to promote maintainable, sustainable research portfolios (DiMasi et al., 2023). With 77% of trial sponsor executives incorporating DCT elements or fully decentralized trials in the next coming years (up from 59% when surveyed in 2021), the time is now to adapt the JTF Core Competency framework to reflect the decentralization of trial activities, organizational system influence on core competencies, and approach CRP skillset through the lens of risk management and safety (LaHucik, 2021).

As demonstrated in this formative study, local clinical providers must be acknowledged and included as partners in trial delivery for participants to be provided research opportunities and for their safety when receiving clinical care. However, suppose providers do not have the skillsets or awareness congruent to those of the CRPs. In that case, challenges will persist with research delivery in communities where trial participation is not the norm. For example, while providers reviewing the patient chart ahead of encounters is a fundamental skill, an advanced skill is identifying that patient as a trial participant. Cultivating provider skillsets as they relate to research is equally important to those of CRPs in rural and frontier areas, given their established trust with the participants as community members but also being the ultimate line of defense related to preventable adverse events (safety) and clinical monitoring. Developing the association of clinical care integration with research activities can be accomplished through enterprise-level leadership and socialization of research programs through grand rounds, organization town halls, and physician-investigator peer mentorship opportunities.

## Conclusion

The call for inclusivity and access equity among rural, frontier, and other under-represented populations also, in turn, means a call to support the recruitment and retention of the clinical research workforce in these areas. The variance in resources, training, and skillsets of CRPs and research program culture across multiple locations necessitates a critical review of organizational culture in clinical care regarding awareness of research activities and their impact on clinical decision-making or risk to patients. The heightened focus on decentralized trial model utilization in rural and frontier areas warrants additional examination and augmentation of the JTF Core Competency Framework to include cultural and resource-contextual considerations aligned to the U.S. Food and Drug Administration decentralized trial guidance. As the results of this formative study highlight, rural research programs need to be integrated with clinical operations to promote awareness and education and foster adaptability across an enterprise as participant and workforce need adjust during this period of rapid change in the industry.

## Data Availability

The raw data supporting the conclusion of this article will be made available by the authors, without undue reservation.
